# Reaction Discovery in Porous Materials Using Periodic Nanoreactor Molecular Dynamics

**DOI:** 10.1002/anie.202514074

**Published:** 2025-12-15

**Authors:** Daniel Deißenbeck, Patrick Meier, Wassja A. Kopp, Anthony D. Debellis, Jan Meisner

**Affiliations:** ^1^ Institute for Physical Chemistry Heinrich Heine University Düsseldorf Universitätsstraße 1 40225 Düsseldorf Germany; ^2^ Quantum Chemistry and Hybrid Modeling Research BASF Corporation Tarrytown NY 10591 USA

**Keywords:** *Ab initio* calculations, Molecular dynamics, Periodic boundary conditions, Selective catalytic reduction

## Abstract

Understanding catalytic processes is essential for advancing energy‐efficient molecular transformations. In heterogeneous catalysis, porous materials such as zeolites play a central role due to their structural complexity and large surface area. Here, we present a periodic ab initio nanoreactor molecular dynamics (NMD) approach to investigate the reaction network of selective catalytic reduction (SCR) of NO over copper‐exchanged chabazite zeolites. This method enables autonomous discovery of both established and previously unreported pathways, including a water‐assisted tautomerization mechanism that facilitates $N_{2}$ formation and a novel radical‐driven route to $N_{2}O$. Notably, NMD simulations also capture reactivity involving Brønsted acid sites of the zeolite framework. By using automated reaction detection, a comprehensive reaction network was constructed, which elucidates the formation of both desired and undesired products. Refinement of the reaction path including free energy corrections by computing the phonon spectrum allows to make quantitative statements about the discovered reactions. The results of this work provide insight into the side‐reactions of SCR, and also demonstrate the versatility of NMD for agnostic reaction discovery of complex systems such as heterogeneous catalysis.

## Graphical abstract

Autonomous reaction discovery using *ab initio* nanoreactor dynamics in combination with periodic density functional theory (DFT) computations elucidates the selective catalytic reduction of NO to $N_{2}$ and side reactions forming $N_{2}O$. This enables the autonomous discovery of reaction mechanisms including the Brønsted sites and proton shuttles, leading to a complex chemical reaction network. Free energy barriers of selected reaction pathways are provided.

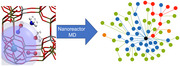

Porous materials such as zeolites are essential in catalysis because their unique structures improve catalytic performance.^[^ ^1^, ^2^, ^3^ ^]^ Due to high surface areas for active sites, molecules can be trapped, and selective interactions with reactants can take place.^[^ ^4^ ^]^ The large complexity of zeolites, in particular when transition metals are involved, allows for a wide variety of possible reaction mechanisms. Using modern tools of computational chemistry, these pathways can be analyzed in depth, and several complex reaction mechanisms could be elucidated,^[^ ^5^, ^6^, ^7^, ^8^, ^9^, ^10^ ^]^ but most of these studies are based on chemical intuition. Methods of autonomous reaction discovery, which agnostically explore possible reaction pathways without relying on predefined mechanistic assumptions, offer a way to overcome this limitation.^[^ ^11^ ^]^ Ideally, reactions could be observed in silico using reactive molecular dynamics (MD) to mimic the actual behavior of the system, but due to the expensive computational costs, reactive MD studies are restricted to time scales of pico‐ to nanoseconds, which is usually insufficient to observe rare reactive events.^[^ ^12^ ^]^ Simply increasing the simulation temperature to accelerate reaction sampling is ineffective, as it can disproportionately favor unimolecular over bimolecular processes.^[^ ^13^ ^]^ To overcome these limitations, alternative strategies are required that can accelerate reaction sampling.^[^ ^14^, ^15^, ^16^, ^17^, ^18^ ^]^ However, most of these techniques require a certain degree of *a priori* insight into the chemical processes and therefore, the outcome can depend on expert knowledge which might introduce a bias. In addition, introduction of a biasing force or energy can change the dynamics of the system and may hinder the direct interpretation of the discovered reactions.^[^ ^19^ ^]^ Different approaches emerged recently which do not require prior knowledge of the chemical mechanism taking place.^[^ ^20^, ^21^ ^]^

One of these methods is *ab initio* nanoreactor molecular dynamics (NMD),^[^ ^22^ ^]^ which accelerates a MD simulation by applying external forces such that even reactions with potential barriers too large to be overcome in regular MD simulations, for example by periodically pushing the molecules toward the center of the system. While this strong compression particularly enhances collisions and bimolecular reactions, other kinds of external forces and, thus, modification of the reactive potential have been shown to accelerate MD for effectively sampling the chemical space.^[^ ^22^, ^23^, ^24^, ^25^, ^26^ ^]^ The settings of the thermostat, such as temperature and friction coefficient, as well as the kind and magnitude of external forces are mere parameters that are only used to enhance reactive events. These parameters need to be chosen system‐dependently to ensure efficient discovery while maintaining the physical meaningfulness, but are not intended to simulate the *operando* conditions, nor to be used to directly calculate physical properties.

The use of external forces, however, introduces a bias of the potential energy surface and therefore changes the dynamics of the system, prohibiting the direct interpretation of the discovered reactions.^[^ ^19^, ^22^ ^]^ Therefore, the idea to separate the generation of reaction mechanism hypotheses from the quantitative evaluation of the reactions emerged.^[^ ^20^, ^27^ ^]^ In this way, first a potential reaction is discovered by NMD, and subsequently, path refinement and transition state optimization techniques are used to obtain data such as reaction energies and barriers.^[^ ^27^ ^]^ NMD reaction discovery still relies on the physical meaningfulness of MD simulations in combination with a potential that qualitatively represents the reactivity of the system. Therefore, the number of occurrences of a particular reaction in NMD simulations can be used as a selection criterion whether reaction pathways should be refined.

In this work, we present a modification of NMD and the application to investigate porous media, in particular zeolite catalysis. For this, density functional theory (DFT) with periodic boundary conditions (PBC) was combined with NMD to discover chemical reaction space. We demonstrate the feasibility of NMD for porous media on the example of selective catalytic reduction (SCR). In SCR, toxic nitrogen oxides carried in exhaust gas are reduced by using ammonia, which is created in situ by thermolysis of an urea solution.^[^ ^28^, ^29^ ^]^ Various catalysts for the SCR have been used^[^ ^6^ ^]^ with copper‐exchanged chabazite being the preferred choice, given its high thermal stability and three‐dimensional porous topology that permits facile diffusion of gas‐phase effluents,^[^ ^30^, ^31^ ^]^ while the large active surface enhances catalytic activity.^[^ ^32^ ^]^ Several mechanisms have already been proposed for the $N_{2}$ formation.^[^ ^33^, ^34^, ^35^, ^36^, ^37^, ^38^ ^]^ The use of DFT in combination with PBCs will allow to discover reactions involving the copper atom, but also Brønsted sites of the framework known to be essential in zeolite catalysis^[^ ^39^ ^]^ without introducing artifacts by truncating the system.^[^ ^5^ ^]^ We therefore pay particular focus on the side reactions of SCR, *i.e*., the formation of the potent greenhouse gas nitrous oxide ($N_{2}O$).^[^ ^37^, ^40^ ^]^

Figure 1a illustrates the concept of NMD simulations for porous media: gas‐phase molecules (here, water ($H_{2}O$), ammonia (${\mathrm{NH}}_{3}$), nitrogen oxide (NO), and molecular oxygen ($O_{2}$)) are inserted into the pore of the copper‐exchanged zeolite ($Z_{2}\mathrm{Cu}$). Details of the unit cell as well as the atoms involved are shown in Figure S1 in the Supporting Information. We have chosen four different initial settings for which five NMD simulations of 20 ps each with random initial configuration were performed, *i.e*., a total of 400 ps simulation time, see Table S1 for further details. The blue and grey spheres in Figure 1 indicate the inner and outer radii $r_{1}$ and $r_{2}$, respectively, outside of which the molecules are pushed toward the origin of the coordinate system by a harmonic bias potential acting on every gas‐phase atom of the system,

(1) $$V_{\text{ext}}=\left\{\begin{array}{cc} \frac{mk_{i}}{2}{\left(r-r_{i}\right)}^{2} & r>r_{i}\\ 0 & r\leq r_{i} \end{array}\right.$$

Here, m is the mass of the atom, r is the atom's distance to the origin of the coordinate system placed close to the position of the copper atom in the crystal structure of the zeolite framework. The force constants $k_{i}$ and $r_{i}$ switch between i=1 in the contraction phase and i=2 in the expansion phase. Figure 1b illustrates the alternating contraction and expansion phases by which gas‐phase molecules are periodically pushed toward the origin of the coordinate system, leading to enhanced reactivity. While restricting the accelerating forces to the gas‐phase molecules keeps the zeolite framework intact, the PBC simulations permit atoms to interact beyond a single cell, leading to discovery largely free from finite‐size effects. This approach avoids unphysical breakage of the zeolite framework and at the same time ensures sufficient reactivity of the gas‐phase species. The periodic DFT calculations use cell parameter, zeolite structure, as well as Γ point calculation from Anggara *et* *al*.^[^ ^41^ ^]^ For all DFT calculations, the riper^[^ ^42^, ^43^, ^44^, ^45^ ^]^ module of the Turbomole programme package^[^ ^46^ ^]^ with the PBE functional^[^ ^47^ ^]^ and SV basis^[^ ^48^ ^]^ was used, see Supporting Information for a more detailed description. Each simulation begins with a $\tau_{1}=500$ fs contraction phase where the gas‐phase molecules are accelerated toward the origin of the coordinate system. The contraction phase is followed by an expansion phase of $\tau_{2}=500$ fs, where the radius of the spherical restraint is enlarged to allow the gas‐phase molecules to diffuse away from the origin of the coordinate system (gray in Figure 1). The time periods $\tau_{1}$ and $\tau_{2}$ were chosen so that most molecules reach the outer region within the expansion phase, enabling them to be accelerated again toward the center when switching again to the contraction phase. This sequence of contraction and expansion phase repeats for the entire simulation time of 20 ps.

**Figure 1:**
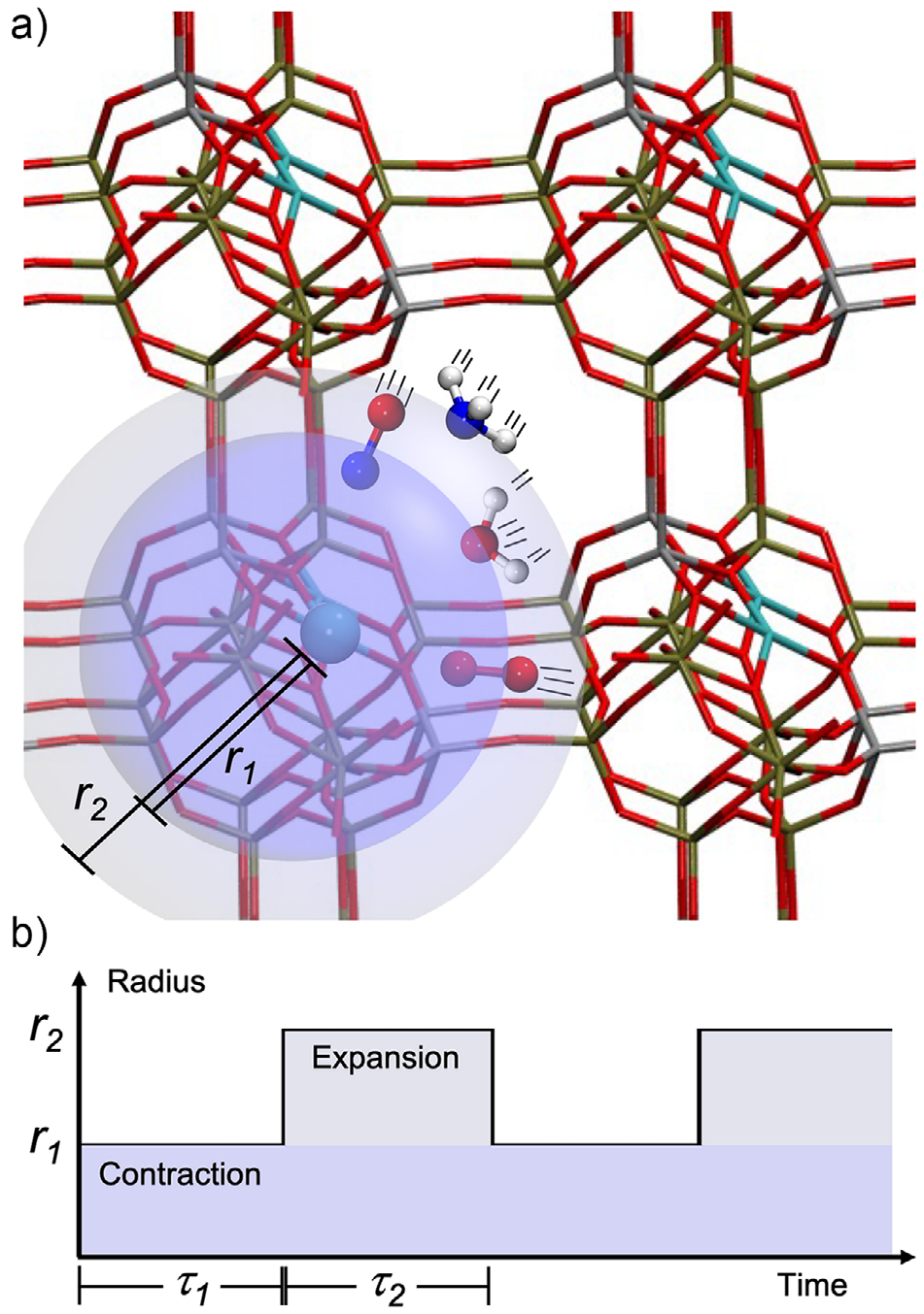
Concept of nanoreactor molecular dynamics in porous media: a) Input molecules shown for the periodic *ab initio* NMD simulations include the copper‐exchanged zeolite cell, ammonia, nitrogen oxide, water, and molecular oxygen. The inner and outer radii $r_{1}$ and $r_{2}$ associated with contraction and expansion phases are represented by blue and light blue spheres, respectively. b) Schematic time plot depicting the switch between contraction phase and expansion phase.

To illustrate dynamics and temperature during such an NMD simulation, an exemplary NMD trajectory is shown in Figure 2. As only the gas‐phase molecules are accelerated during the contraction phase, the temperature of the total system stays close to the temperature of the Langevin thermostat^[^ ^49^ ^]^ of 1500 K. As mentioned above, the temperature used in NMD simulations serves as a parameter to ease chemical reactivity. In the particular NMD trajectory shown in Figure 2 starting with 2 NO, 2 ${\mathrm{NH}}_{3}$, and one $O_{2}$ molecules (Setting #2, Run 3, see Table S1), the initial contraction leads to four elementary reactions within the first 500 fs, without destroying the zeolite framework: an NO radical adsorbs onto the Cu(II) ion and is then subject to nucleophilic attack by an ammonia molecule. This finding aligns with previous literature stating that NO coordination *via* the nitrogen atom to the copper (II) ion is preferred over coordination *via* the oxygen atom.^[^ ^50^ ^]^ Subsequently, a second ammonia molecule deprotonates the intermediate complex, forming a coordinated nitrosamine ($H_{2}\mathrm{NNO}$). In this NMD trajectory, the nitrosamine molecule stays bound to the copper atom for around 16 ps, which indicates that this species is less reactive and comparatively stable. In other NMD trajectories we find that the deprotonation step can occur *via* a Brønsted site of the zeolite (see Figure S3 in the Supporting Information), which is in accordance with Mao *et* *al*.^[^ ^50^ ^]^ We also observed the formation of a ${[Z}_{2}\mathrm{Cu}$]─${\mathrm{NH}}_{3}$ complex and a complex where both ${\mathrm{NH}}_{3}$ and NO are adsorbed, ${[Z}_{2}\mathrm{Cu}$]─(NO)(${\mathrm{NH}}_{3}$), in NMD simulations (e.g., Setting #1, Run 2 and Setting #1, Run 1, respectively). As pointed out by Mao *et* *al*., these complexes can also form nitrosamine, which was, however, not observed in our simulations.^[^ ^50^ ^]^

**Figure 2:**
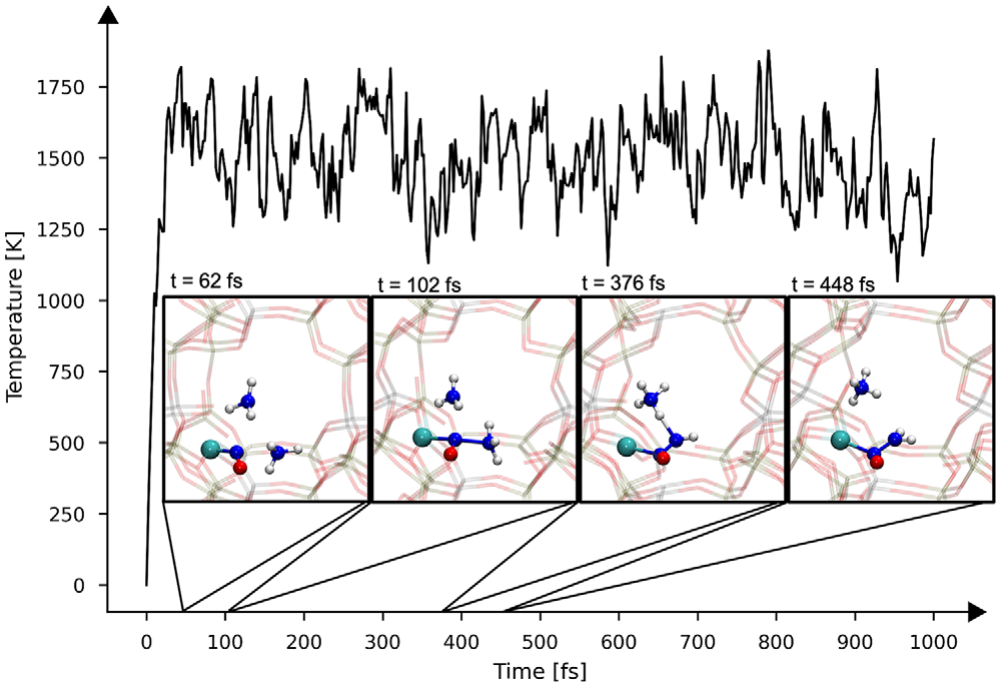
Temperature during a typical NMD discovery simulation starting with 2 NO, 2 ${\mathrm{NH}}_{3}$, $O_{2}$, and the zeolite framework (Setting #2, Run 3; zeolite framework is in transparent and only reacting molecules are shown for clarity). The simulation started with the contraction phase, which triggered chemical reactions leading to chemisorbed nitrosamine ($H_{2}\mathrm{NNO}$) already in the first few hundred steps.

In addition to visual inspection of the NMD trajectories, we applied an automated reaction detection scheme to systematically identify all observed reactions. Automated analysis of reactivity in molecular dynamics can be based on either interatomic distances or bond order metrics. In this work, we used Wiberg bond orders,^[^ ^51^ ^]^ which can be computed on‐the‐fly during the simulation. Reactions are detected by monitoring bond order changes using a two‐threshold criterion, and all identified events are compiled into a reaction network. This approach enables efficient reaction discovery while allowing the network to be refined later to limit computational cost. Further details on the detection scheme and thresholds are provided in the Supporting Information (Section S3). In the 400 ps NMD simulation time, 1703 unique species and 1548 unique reactions were detected, which corresponds to a discovery rate of 3.87 reactions per ps. These reactions are used to construct a chemical reaction network, see Figure 3. However, several reactions where Si─O or Al─O bonds are broken (most of them temporarily) led to a large number of rearrangement reactions of the zeolite. For example, the reversible initial reaction step of the hydrolysis of an Al─O bond by insertion of a water molecule was observed (see Figure S9).^[^ ^52^ ^]^ However, to detect irreversible framework degradation, more water molecules would have to be included. In this work, we want to focus on the catalytic reactivity of the undamaged zeolite rather than the degradation of the zeolite framework. Accordingly, we restricted the reaction network to the reactions where gas‐phase molecules react with the zeolite or already adsorbed gas‐phase molecules react, which leads to a number of 225 unique species and 370 unique reactions.

**Figure 3:**
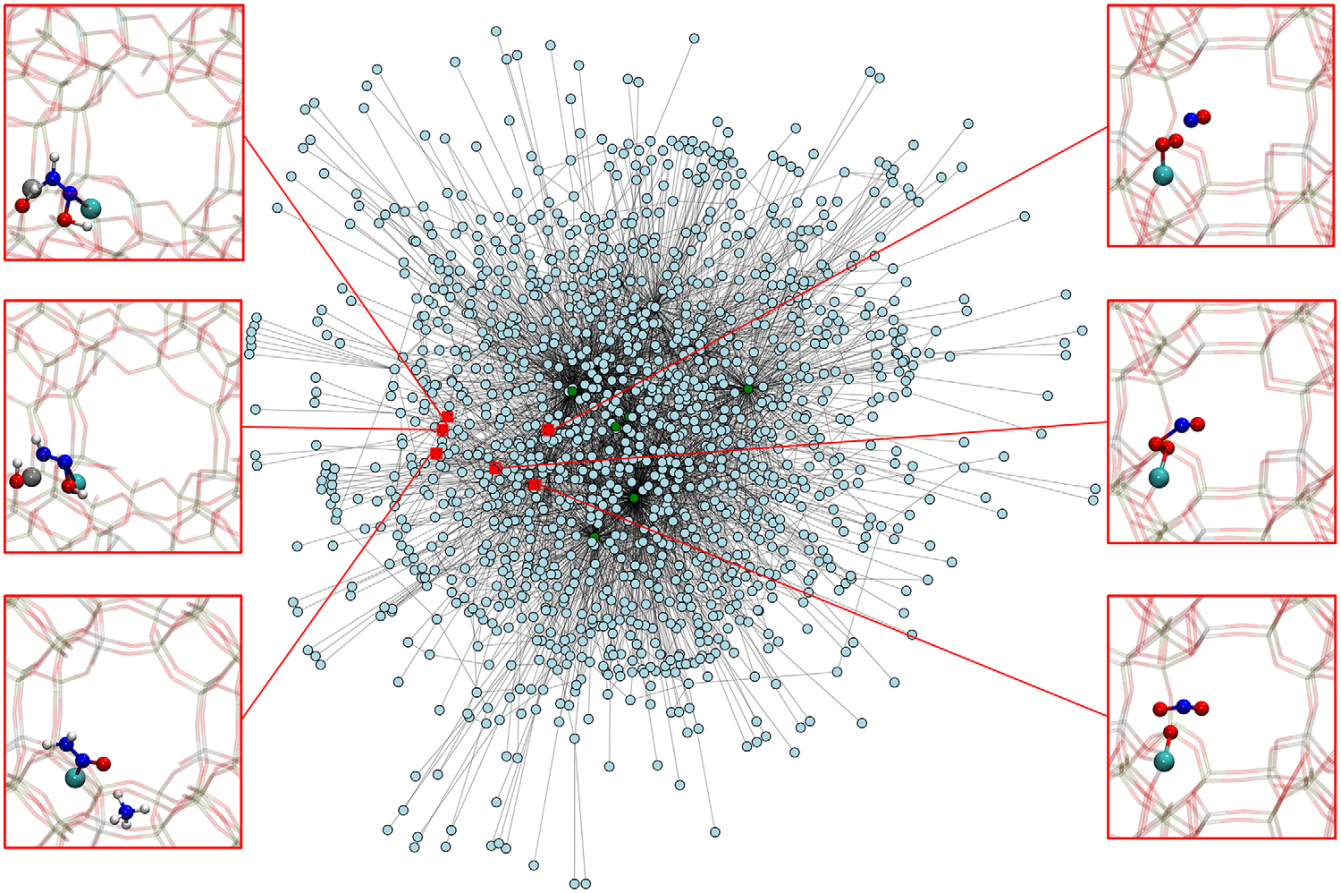
Reaction network generated from 400 ps of NMD simulations with copper‐exchanged zeolite, NO, ${\mathrm{NH}}_{3}$, $H_{2}O$, and $O_{2}$ as initial species (green nodes), see settings #1–4 in Supporting Information. Exemplary species and reactions illustrate key mechanistic features: (top and middle left) Brønsted acid sites in the zeolite framework actively participate in proton transfer reactions, including deprotonation of coordinated $H_{2}\mathrm{NNOH}$; (bottom left) formation of ${[Z}_{2}\mathrm{Cu}$]─$H_{2}\mathrm{NNO}$ as a stable intermediate; (right) copper‐catalyzed oxidation of NO to ${\mathrm{NO}}_{2}$ by $O_{2}$, consistent with Mao *et* *al*.^[^ ^50^ ^]^

Apart from the formation of nitrosamine, one of the main intermediates of SCR according to the literature,^[^ ^8^, ^50^, ^53^, ^54^, ^55^ ^]^ we have observed interesting side reactions of the adsorbed molecules with each other, but also with the zeolite framework. During our simulations, multiple proton transfer events occur involving the zeolite framework through its Brønsted acid sites, which are known to facilitate protonation and deprotonation of molecules and intermediates.^[^ ^56^ ^]^^[^ ^57^, ^58^, ^59^ ^]^ Due to the agnostic nature of NMD, such reactions involving the zeolite framework can naturally emerge during the simulations. As illustrated in Figure 3 (left), a protonated nitrosamine coordinated to the copper atom (${[Z}_{2}\mathrm{Cu}$]─$H_{2}\mathrm{NNOH}$) is shown to transfer its proton to a framework oxygen atom bonded to an aluminum atom in the zeolite, resulting in ${[Z}_{2}\mathrm{Cu}$]─HNNOH. These representative snapshots underscore the Brønsted activity of the zeolite framework and highlight its dynamic role in the facilitation of proton shuttle reactions. Furthermore, we observed the coordination of the copper atom by ammonia molecules, solvating the copper atom as [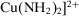 complex, which is a well‐known phenomenon.^[^ ^54^, ^60^, ^61^, ^62^ ^]^ Besides ${\mathrm{NH}}_{3}$ molecules, our NMD simulations also show water molecules mobilizing the copper ion. Additionally, we found a literature‐known copper‐catalyzed formation mechanism of ${\mathrm{NO}}_{2}$ radicals from NO and $O_{2}$, reported by Mao *et* *al*.^[^ ^50^ ^]^ and Chen *et* *al*.^[^ ^63^ ^]^ (Figure 3). Finding these diverse side reactions highlights the capabilities of our method for autonomous reaction network construction for a system as complex as copper catalysis within zeolites.

Given the central role of coordinated nitrosamine as a key intermediate, we conducted additional NMD simulations starting from configurations in which nitrosamine is bound to the copper center, *i.e.*, ${[Z}_{2}\mathrm{Cu}$]─$H_{2}\mathrm{NNO}$ (again five runs of 20 ps each; see settings #5 and #6 in the Supporting Information). In some of these NMD simulations, ${\mathrm{NO}}_{2}$ was added because it also was discovered to be formed in some trajectories (see Figure 3, right snapshots), which confirmed its relevance and prompted us to include this species. We also performed additional NMD simulations with water in the presence of ${[Z}_{2}\mathrm{Cu}$]─$H_{2}\mathrm{NNO}$. By adding ${\mathrm{NO}}_{2}$ radicals, we discovered a previously unreported intermediate species resulting from H atom abstraction of the coordinated nitrosamine,

$$\left[Z_{2}\mathrm{Cu}\right]-H_{2}\mathrm{NNO}+{\mathrm{NO}}_{2}⟶\left[Z_{2}\mathrm{Cu}\right]-\mathrm{HNNO}+\mathrm{HONO}.$$

This led us to initialize further NMD simulations starting from this newly discovered adsorbed HNNO intermediate (five simulations of 20 ps simulation time each, see setting #7 in the Supporting information). Starting from ${[Z}_{2}\mathrm{Cu}$]─HNNO, further H atom abstraction of another ${\mathrm{NO}}_{2}$ radical forms another HONO molecule and leads to $N_{2}O$ coordinated to the copper atom,

$$\left[Z_{2}\mathrm{Cu}\right]-\mathrm{HNNO}+{\mathrm{NO}}_{2}⟶\left[Z_{2}\mathrm{Cu}\right]-\mathrm{NNO}+\mathrm{HONO}$$

which eventually dissociates, releasing Cu(I). This radical reaction mechanism, which is shown in Figure 4, can in principle also occur with other radicals such as OH, which are formed during combustion, but we limit ourselves to the reaction with ${\mathrm{NO}}_{2}$ molecules.

**Figure 4:**
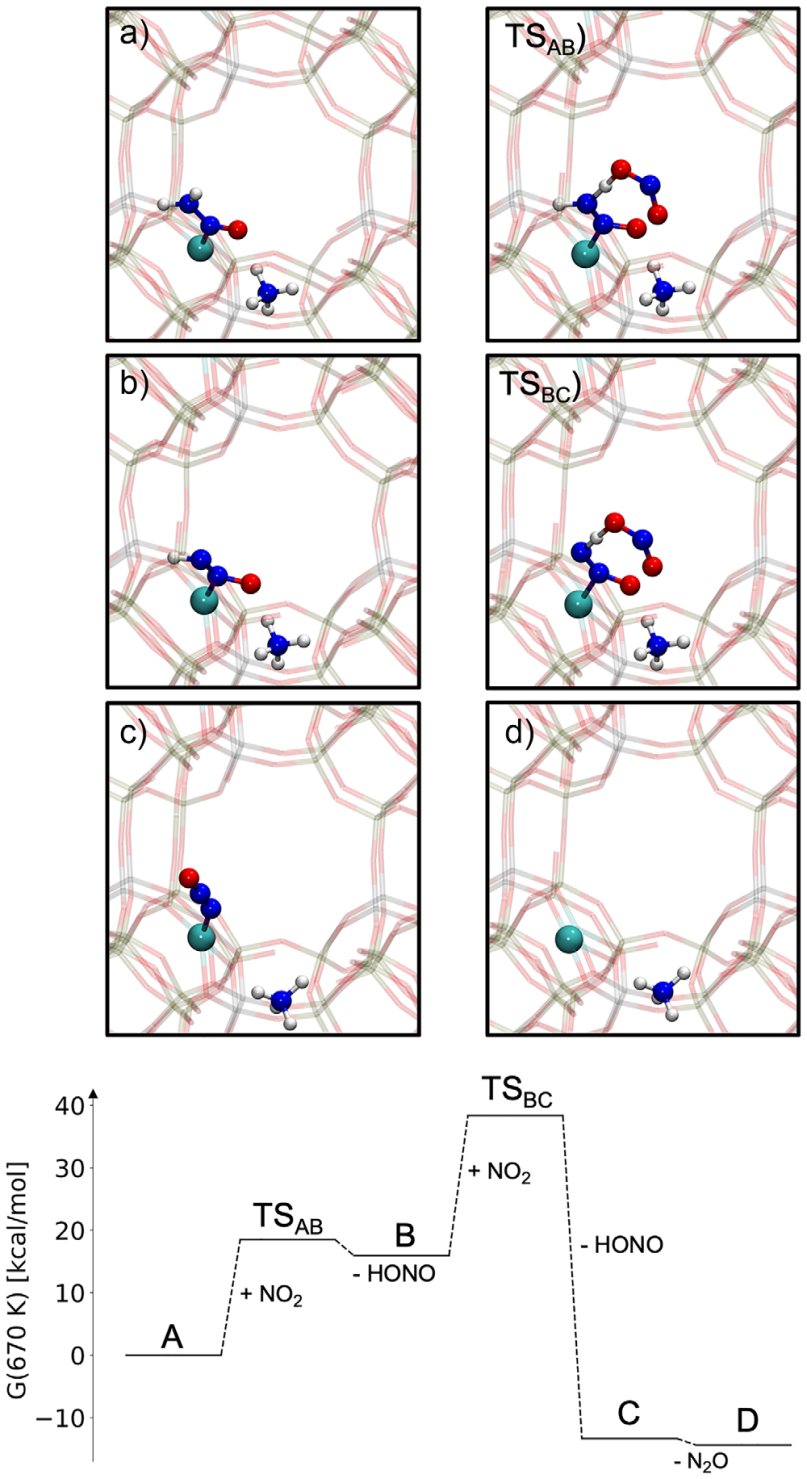
Refined reaction mechanism of the $N_{2}O$ formation as discovered in the NMD simulations. Top: molecular structures where the involved atoms are highlighted. An ${\mathrm{NH}}_{4}$ ion, which was formed during the ${[Z}_{2}\mathrm{Cu}$]─$H_{2}\mathrm{NNO}$ formation,‐ was placed in the pore to maintain charge neutrality.

In order to obtain insight into the feasibility of the novel $N_{2}O$ formation mechanism, we refined the associated elementary reaction steps. We focused on pathways where nitrosamine is involved, as these are likely to play a key role in the main SCR mechanism as well as in the $N_{2}O$ forming side‐reaction. Due to the high computational cost, we limited the refinement process to reactions that recurred across independent trajectories in order to deprioritize one‐off events. Ultimately, the mechanistic picture is not based on the frequency of a reaction in NMD trajectories, but on the energetic evaluation of the individually refined reaction pathways.

For this refinement, we used a modified version of Chemshell^[^ ^64^ ^]^ interfaced to Turbomole to optimize the corresponding minimum and localize transition state structures. The PBE functional and def2‐SVP^[^ ^65^ ^]^ basis were used to obtain stationary points and energy corrections with the HSE06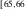 ^]^ functional and the def2‐SVP basis set were performed. As bimolecular reactions are involved, thermal contributions such as entropy need to be taken into account for a proper description of free energy barriers. Hence, we computed acoustic and optical phonon vibrations using Phonopy^[^ ^66^, ^67^ ^]^ with the frozen phonon approach. Free energies and barriers are evaluated at 670 K. Further information can be found in the Supporting Information Section S5.

For the first H atom abstraction, we obtained a free energy barrier of 18.5 kcal ${\mathrm{mol}}^{-1}$ with respect to ${[Z}_{2}\mathrm{Cu}$]─$H_{2}\mathrm{NNO}$ and ${\mathrm{NO}}_{2}$ separated. A more detailed version of this mechanism including the pre‐ and post‐reactive complexes of the elementary steps can be found in the Supporting Information (Figure S12). Upon formation of ${[Z}_{2}\mathrm{Cu}$]─HNNO, the free energy increases by 15.9 kcal ${\mathrm{mol}}^{-1}$ as the formed HONO molecule leaves the system. For the second H atom abstraction, we obtained a reaction barrier of 22.4 kcal ${\mathrm{mol}}^{-1}$. This reaction is exothermic with a reaction free energy of –29.3 ${\mathrm{kcalmol}}^{-1}$, as only closed‐shell systems, *i.e*., ${[Z}_{2}\mathrm{Cu}$]─$N_{2}O$ and HONO, result. All these free energy barriers are achievable at an *operando* temperature of 400 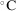. Thus, once ${[Z}_{2}\mathrm{Cu}$]─$H_{2}\mathrm{NNO}$ is formed and a certain concentration of radicals is present, $N_{2}O$ can be formed in an exothermic reaction with free reaction energy of ‐14.4 $\mathrm{kcal}\;{\mathrm{mol}}^{-1}$.

NMD is known to strongly promote bi‐ and termolecular reactivity due to the strong compressive forces fostering collisions so that reaction barriers can be overcome. In contrast, unimolecular reactions are not accelerated as much and therefore, our NMD discovery simulations missed the intramolecular [1,3] H shift of $H_{2}\mathrm{NNO}$ to the tautomer HNNOH, which is known to be essential for $N_{2}$ formation.^[^ ^8^, ^50^, ^53^, ^54^, ^68^, ^69^, ^70^ ^]^ However, the addition of water molecules in our NMD simulations revealed a water‐shuttled tautomerization of the Cu‐coordinated nitrosamine, similar to the previously reported water‐shuttled mechanism in the gas‐phase, see Figure 5.^[^ ^71^ ^]^ A comparison of the free energy barriers of 36.3 $\mathrm{kcal}\;{\mathrm{mol}}^{-1}$ for the plain [1,3] H shift with 11.7 $\mathrm{kcal}\;{\mathrm{mol}}^{-1}$ for the water‐shuttled [1,3] H shift reveals a significant catalytic effect of water on this mechanism, see Supporting Information Figure S13. The catalytic effect of water molecules in the zeolite pores is in accordance with experimental findings that water can indeed improve the SCR.^[^ ^72^ ^]^ In the gas phase, water can mediate nitrosamine decomposition by utilizing a shuttle mechanism, reducing the barrier to 11.5 $\mathrm{kcal}\;{\mathrm{mol}}^{-1}$.^[^ ^71^ ^]^ The subsequent reaction of ${[Z}_{2}\mathrm{Cu}$]─HNNOH to $N_{2}$ and $H_{2}O$ is presumed to take place in the gas phase or at a Brønsted site.^[^ ^50^ ^]^

**Figure 5:**
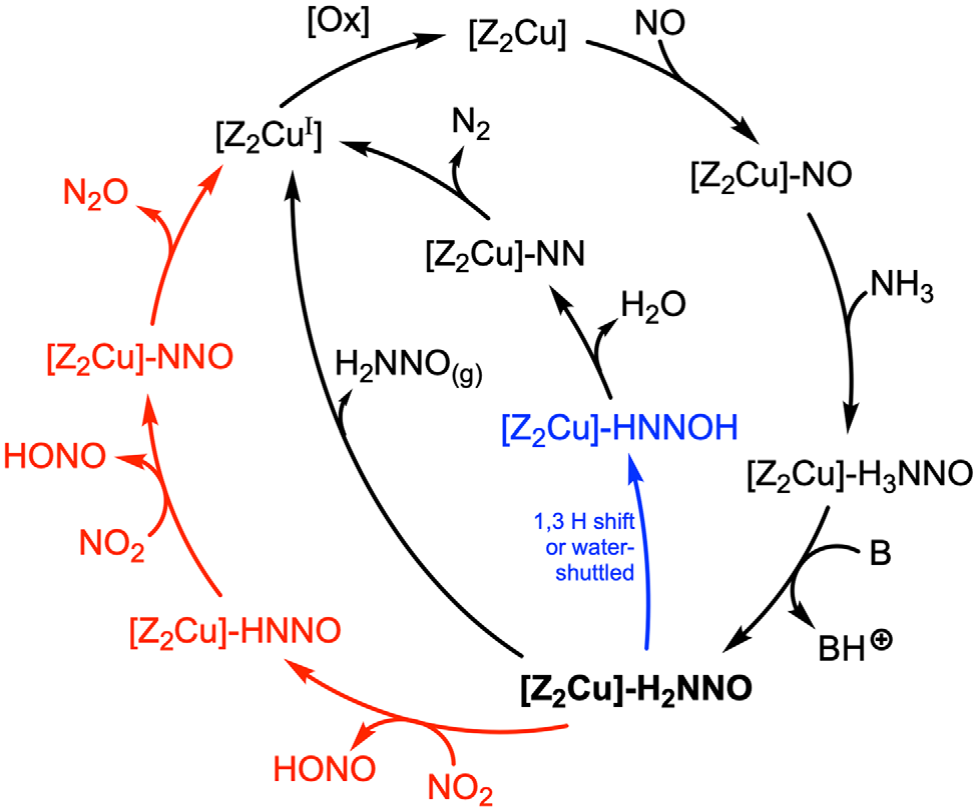
Comprehensive catalytic cycle for Cu‐catalyzed SCR as discovered through NMD and metadynamics simulations. The deprotonation of ${[Z}_{2}\mathrm{Cu}$]─$H_{3}\mathrm{NNO}$ can be performed by a base (*e.g*., ${\mathrm{NH}}_{3}$) or an oxygen atom from the zeolite framework (see Figure S3). Two reaction mechanisms are included here: (black) the established sequence leading to $N_{2}$ and $H_{2}O$ *via* nitrosamine formation (${[Z}_{2}\mathrm{Cu}$]─$H_{2}\mathrm{NNO}$) and decomposition, including a water‐assisted [1,3] hydrogen shift (blue); and (red) a newly discovered radical‐mediated pathway resulting in $N_{2}O$ formation through sequential H atom abstraction by ${\mathrm{NO}}_{2}$. The oxidative half‐cycle^[^ ^8^ ^]^ ([Ox]) is indicated but not directly explored in this study. The central intermediate ${[Z}_{2}\mathrm{Cu}$]─$H_{2}\mathrm{NNO}$ serves as a branching point, highlighting how the total reaction mechanism is influenced by the concentrations of gas‐phase molecules.

Considering the two possible reaction pathways, resulting in $N_{2}$ and $N_{2}O$, respectively, it becomes evident that nitrosamine, coordinated to the copper atom as ${[Z}_{2}\mathrm{Cu}$]─$H_{2}\mathrm{NNO}$, plays a central role as a key branching point in the overall catalytic process (highlighted as bold in Figure 5). If nitrosamine dissociates, it decomposes to $N_{2}$ and $H_{2}O$, further catalyzed by water, as investigated by Chen *et* *al*.^[^ ^71^ ^]^ If nitrosamine stays adsorbed, also oxidation to ${[Z}_{2}\mathrm{Cu}$]─HNNO and further to $N_{2}O$ can occur (marked in red in Figure 5). We therefore suggest that our composed mechanistic cycle explains a varying reactivity due to different reaction conditions, *i.e*., enhanced $N_{2}O$ formation under oxidative conditions (in particular a high radical concentration) or increased $N_{2}$ formation in particular when more water molecules are available. Nitrosamine as central intermediate can also be formed when either ${\mathrm{NH}}_{3}$ is adsorbed first or when both ${\mathrm{NH}}_{3}$ and NO are adsorbed,^[^ ^50^ ^]^ which would change the order of the first two steps starting from ${[Z}_{2}\mathrm{Cu}$] in Figure 5.

The [1,3] H shift leading to ${[Z}_{2}\mathrm{Cu}$]─HNNOH formation, which escaped NMD discovery, was indeed found by running RMSD‐based metadynamics^[^ ^16^ ^]^ simulations starting from ${[Z}_{2}\mathrm{Cu}$]─$H_{2}\mathrm{NNO}$. RMSD‐based metadynamics tends to find reactions with lower energy barriers which are entropically favored^[^ ^25^ ^]^ and is therefore complementary to the periodically compressing NMD simulations which efficiently explores bimolecular reactivity.^[^ ^22^ ^]^ Again, five trajectories of 20 ps each have been initialized, demonstrating that MD‐based reaction discovery is able to also find this reaction step (see Supporting Information for further details and Figure S8 for snapshots of this reaction mechanism). Noteworthily, in our simulations $N_{2}$ was formed *via* a framework‐assisted shuttle mechanism within the zeolite, rather than the plain, literature‐known mechanism.^[^ ^50^, ^68^ ^]^ A further literature‐known $N_{2}O$ formation mechanism is the decomposition of ammonium nitrate.^[^ ^34^ ^]^ There is evidence that this mechanism is not the major reaction mechanism and alternative mechanisms are taking place.^[^ ^37^ ^]^ In our NMD and metadynamic simulations neither the formation of ammonium nitrate, nor the decomposition thereof was observed.

In summary, *ab initio* NMD with PBCs was used to autonomously discover the chemical reaction network of SCR starting from simple initial molecules inserted into the pore of a copper‐exchanged chabazite. Re‐introducing species obtained from simulations as initial reactants, in particular ${[Z}_{2}\mathrm{Cu}$]─$H_{2}\mathrm{NNO}$, expanded the reaction network and explored chemical reaction space further. In total, 800 ps of MD‐based reaction discovery was performed, leading to 3050 unique species and 2810 unique reactions. A visualization of the complete reaction network is shown in the Supporting Information (Figure S11). The MD‐based reaction discovery performed in this work found established reaction pathways of the main $N_{2}$ formation mechanism of SCR but also novel side‐reactions. From the reaction hypotheses created in these discovery simulations, the ones recurring across multiple NMD simulations and involving key chemical species were refined. Hence, the mechanistic conclusions in this work rely on refined pathways with HSE06 single‐point energies and phonon‐based free‐energy corrections evaluated at 670 K.

Our results showed that a water‐shuttled mechanism significantly reduces the activation free energies of the formation of $N_{2}$, which explains the previously observed catalytic effect of water on the SCR.^[^ ^73^ ^]^ Even more interestingly, a novel formation mechanism of the highly potent greenhouse gas $N_{2}O$ via a radical reaction pathway was discovered, which explains the influence of ${\mathrm{NO}}_{2}$ on unwanted $N_{2}O$ formation during SCR.^[^ ^74^ ^]^ Our discovered reaction pathway presented here may be a feasible alternative mechanism if a sufficient amount of radicals is available. The identification of a novel radical‐mediated pathway for $N_{2}O$ formation offers valuable mechanistic insight into side reactions in SCR catalysis. Understanding this route is particularly relevant for the rational design of catalysts that minimize undesired $N_{2}O$ emissions—a major environmental concern due to its potency as a greenhouse gas. Our findings suggest that adding water to the exhaust gas and controlling the radical concentration may indeed improve SCR efficiency in future catalyst development. However, more extensive simulations including the construction of a more dense chemical reaction network would be necessary to elucidate the contribution of each single $N_{2}O$ formation mechanism and account for the interplay of the different, competing mechanisms. The methodology presented here can also be used to construct reaction networks of other, similarly complex chemical systems such as interfaces and porous media.

## Conflict of Interests

The authors declare no conflict of interest.

## Supporting information

Supporting Information

## Data Availability

The data that support the findings of this study are openly available in Zenodo at https://doi.org/10.5281/zenodo.15737638, reference number 15737639.
