# Supplementary material for: Reaction Discovery in Porous Materials Using Periodic Nanoreactor Molecular Dynamics
Source: Angew Chem Int Ed Engl. 2025 Dec 15;65(6):e14074. doi: 10.1002/anie.202514074 (PMC12865154; doi:10.1002/anie.202514074)
Supplement: Supplementary file 1 — Supporting Information [file ANIE-65-e14074-s001.pdf]

Angewandte Chemie
Eine Zeitschrift der Gesellschaft Deutscher Chemiker

Supporting Information

Reaction Discovery in Porous Materials Using Periodic Nanoreactor Molecular Dynamics

Daniel Deißenbeck, Patrick Meier, Wassja A. Kopp, Anthony D. Debellis, Jan Meisner*

Contents

1. Reaction Discovery Setup **3**

2. Additional Information for the Mechanisms Discussed **5**

2.1. Snapshots of Exemplary Deprotonation by a Brønsted Site 5
2.2. Snapshots of the Cu-catalyzed NO_2 Formation Mechanism 5
2.3. Snapshots of the N_2O Formation Mechanism 6
2.4. Snapshots of the H_2O-shuttled mechanism of nitrosamine 6
2.5. 1,3-H-shift mechanism snapshots 7
2.6. Snapshot of water insertion into the zeolite framework 8

3. Bond Detection **9**

4. Reaction Network **10**

5. Refinement of Reaction Paths **11**

5.1. N_2O formation mechanism 11
5.2. H_2O-shuttled reaction mechanism 13

1. Reaction Discovery Setup

Ab initio nanoreactor[1] molecular dynamics (NMD) were interfaced with periodic boundary conditions from the riper module[2–5], which is part of Turbomole Version 7.7[6] All discovery trajectories start with the zeolite cell (Z_2Cu) with the cell parameters (a = 9.409 Å, b = 9.444 Å, c = 9.381 Å) taken from Anggara et al.[7]. The structure includes 12 tetrahedral (T)-sites, made of 37 atoms in total (24 O atoms, 10 Si atoms, 2 Al atoms and one Cu atom). The calculations were performed using a single unit cell as Γ point calculations, see Fig. S1. The gas phase input molecules were packed into a sphere with a radius of 4.0 Å and centered at (4.0 Å, 4.5 Å, 4.0 Å) using the packmol program. All runs were performed using velocity Verlet integration with a time step of 2 fs. To address discretization issues related to hydrogen atoms, the mass of hydrogen atoms has been doubled. Feenstra et al. showed that increasing the time step and the hydrogen mass to 2 leads to enhanced simulation time without significantly decreasing accuracy.[8]

A Langevin thermostat[9] with a friction term of 41 ps^{-1} and a temperature of 1500 K was employed. As described in the main text, four settings (#1-4, see Table S1) have been chosen. Nitrogen oxide (NO), ammonia (NH_3), water (H_2O), and molecular oxygen (O_2) have been used as input molecules. To explore complementary regions of chemical space, simulations were also started from the nitrosamine (H_2NNO) bound to the copper atom ([Z_2Cu]-H_2NNO) and H_2O and NO_2 as further input molecules (#5 and #6). The formation of H_2NNO is accompanied by the simultaneous generation of an ammonium cation. Accordingly, in the runs starting from [Z_2Cu]-H_2NNO, the ammonium cation was added, maintaining charge neutrality. Additional simulations were also started from the discovered nitrosaminyl radical (HNNO) bound to the copper atom ([Z_2Cu]-HNNO) and NO_2 (#7).

These simulations were conducted over a simulation time of 20 ps (see Table S1). To enhance rare event sampling, two complementary methods of molecular discovery were used, a compressing spherical potential and root mean square deviation (RMSD)-based metadynamics[10]. The RMSD-based metadynamics[10] simulations, which were initialized starting from H_2NNO with a temperature of 1500 K, were run for a simulation time of 20 ps. Metadynamics settings include a strength k_i = 250 kcal/mol, a frequency with which new reference structures are added (τ_{mtd} = 1000 fs^{-1}) and a damping factor of κ = 0.03. See the original reference 10 for further methodological details.

Table S1. List of settings regarding the *ab initio* molecular dynamics simulations for the database, with the compressing force settings and their input molecules each setting was performed with 5 different starting structures and 20 ps simulation time.

Simulation Settings	r_1 [Å]	k_1 $\left[\frac{\mathrm{kcal}}{\mathrm{mol \cdot Å^2}}\right]$	r_2 [Å]	k_2 $\left[\frac{\mathrm{kcal}}{\mathrm{mol \cdot Å^2}}\right]$	Input Molecules
	Compressing force				
1	4.8	0.45	7.9	0.22	2 NO, 2 NH_3, 2 H_2O
2	4.8	0.45	7.9	0.22	2 NO, 2 NH_3, O_2
3	4.8	0.45	7.9	0.22	2 NO, NH_3, H_2O, O_2
4	4.8	0.75	7.9	0.25	2 NO, NH_3, H_2O, O_2
5	4.8	0.75	7.9	0.25	2 H_2O, NH_4^+, H_2NNO
6	4.8	0.45	7.9	0.22	3 NO_2, NH_4^+, H_2NNO
7	4.8	0.45	7.9	0.22	2 NO_2 NH_4^+, HNNO

The PBE[11] functional with D3 dispersion correction[12] and the SV basis set[13] with a def2-SV(P)[14] auxiliary basis set was used to compute the reactive potential for all *ab initio* nanoreactor simulations. A Self-consistent field (SCF) convergence threshold of 10^{-4} and the DFT grid of m5 was employed. To ensure SCF convergence stability, damping of a maximum of 15.0, with a stepwise increment of 0.1 and minimum value of 0.5 was used. Additionally, an orbital shift of 0.1 and Fermi smearing with a temperature of 3000 K were implemented. In case of SCF failure, initially backup options with a damping maximum of 25 and a higher max iteration of 1000 and post-backup options with a damping maximum of 50 and a higher max iteration of 10000 have been used to ensure convergence on the cost of large computational time.

In our NMD simulations, a periodically compressing force (see Eq. 1 in the main text) is used to enhance collisions. If an atom is outside of the radius r_1 (inner radius) or r_2 (outer radius), it will pushed towards to origin of the coordinate system using a harmonic potential with the force constants k_1 and k_2 during the contraction or expansion phase, respectively. Only the gas-phase molecules are subjected to this accelerating force in order to maintain the zeolite framework intact. Contraction and expansion phase both last 500 fs.

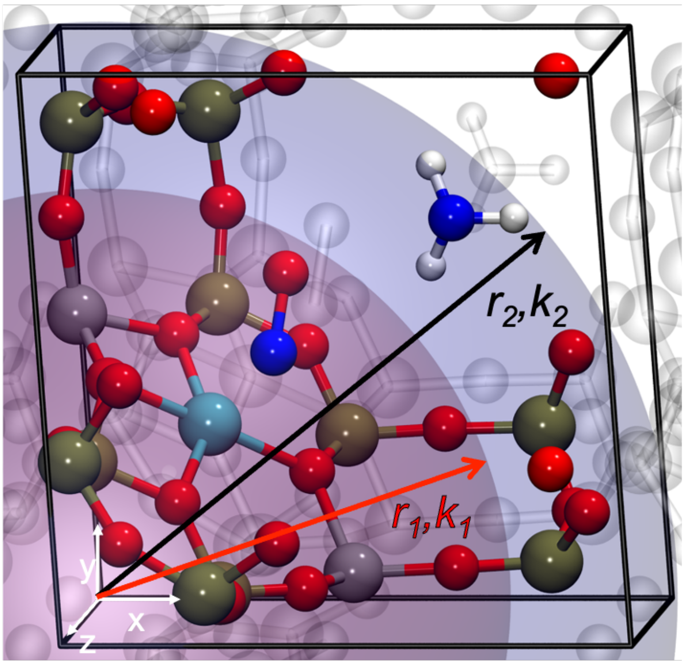

Figure S1. The unit cell of the Cu-SSZ-13 zeolite together with one NO and NH_3 as example input molecules are shown. Surrounding zeolite cells are indicated as transparent structures, while the zeolite framework is shown with the copper atom (cyan), aluminum atoms (grey) as Brønsted sites, silicon atoms (brown) and oxygen atoms (red). The origin is indicated by the white Cartesian coordinate system. Sphere radius for the contraction phase in red (r_1) and the expansion phase in black (r_2) centered to the origin with their corresponding force constants are colored as red and blue spheres.

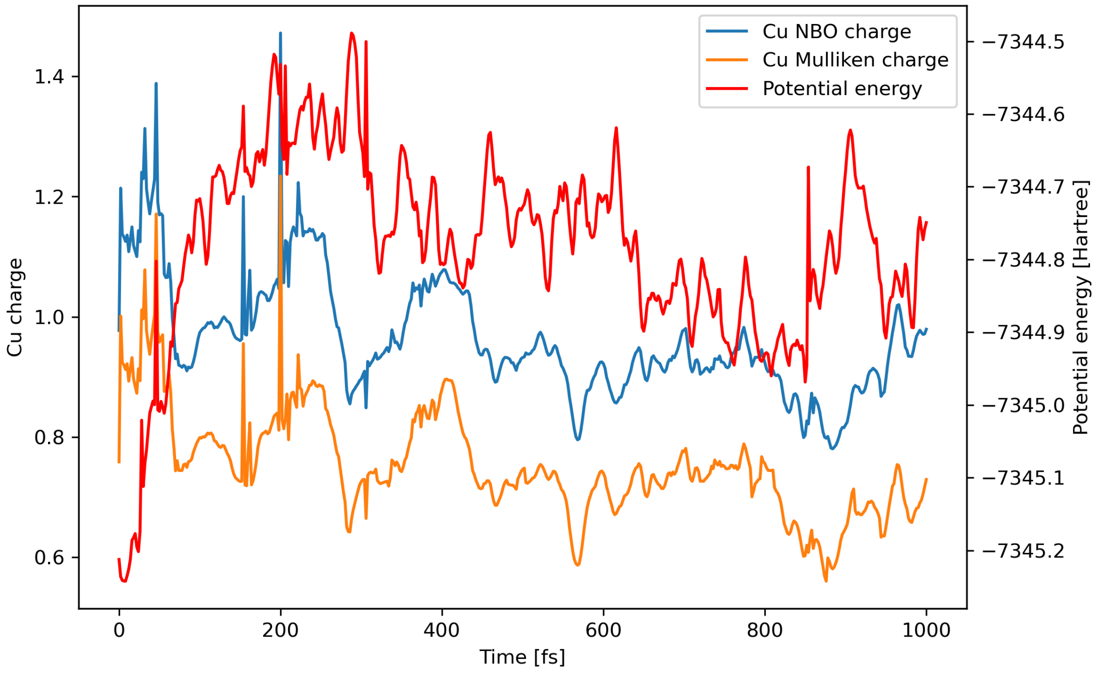

Figure S2. NBO[15] and Mulliken[16] charges along an NMD trajectory (Setting #1 Run Nr. 2). Even though the NMD trajectories have large-amplitude motion due to the high temperature and external forces (which can be seen by the large fluctuations in the potential energy of the trajectory) the NBO charge of the Cu atom does not change by more than 0.2.

2. Additional Information for the Mechanisms Discussed

Various reactions which are discussed in the main text are shown in further detail below. In the trajectories with the settings # 1 to 4, we observed reactions involving Brønsted centers (Fig. S3) and the formation of NO_2 (Fig. S4). Trajectories with the setting # 5, i.e. starting from [Z_2Cu]-H_2NNO and NO_2, obtained a novel reactions mechanism for N_2O formation. See Fig. S5 and Fig. S6 depicting snapshots along the trajectories and Fig. 4 in the main text for the structures of the refined mechanism. NMD reaction discovery starting from [Z_2Cu]-H_2NNO and H_2O resulted in a water-shuttled mechanism (Fig. S7). RMSD-based metadynamics gave a 1,3 H shift (Fig. S8). After performing these discoveries we have automatically detected reactions by using bond order detection.

2.1. Snapshots of Exemplary Deprotonation by a Brønsted Site

The Brønsted sites of the zeolite framework deprotonated various species during the NMD simulations. As an exemplary deprotonation reaction, the proton transfer from the [Z_2Cu]-H_3NNO to a Brønsted site of the zeolite framework forming nitrosamine ([Z_2Cu]-H_2NNO) is shown in Figure S3.

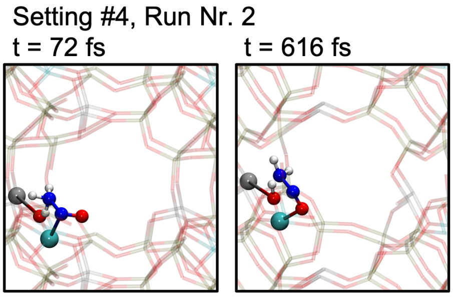

Figure S3. Snapshots of the deprotonation of [Z_2Cu]-H_3NNO by a Brønsted site of the zeolite framework resulting of nitrosamine coordinated to the Cu atom, i.e. [Z_2Cu]-H_2NNO. Atoms participating in this reaction before (left) and after (right) the proton transfer are highlighted, other atoms are shown in the background. Color scheme is identical to Fig. S1.

2.2. Snapshots of the Cu-catalyzed NO_2 Formation Mechanism

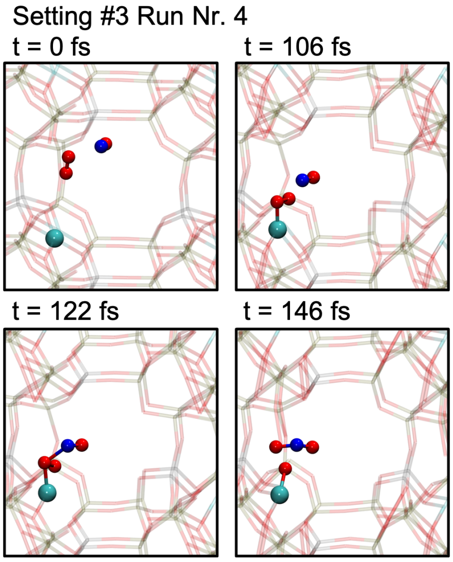

Figure S4. Snapshots of the Cu-catalyzed oxidation of NO with O_2 as oxidant (Color scheme is identical to Fig. S1.) Same color scheme and visualization setting as Fig. S3 Snapshots of the NO_2 formation: Upper left: start of the discovery trajectory with Z_2Cu, O_2, and NO. Upper right: coordination of the O_2 to the Cu atom. Lower left: abstraction of an oxygen atom by the incoming NO radical. Lower right: formation of the NO_2 and the remaining oxygen atom bound to the copper atom.

2.3. Snapshots of the N_2O Formation Mechanism

Starting from [Z_2Cu]-H_2NNO and NO_2 as input molecules (setting # 6), revealed the H atom abstraction mechanisms depicted in Figure S5 shows the formation of the [Z_2Cu]-HNNO starting from [Z_2Cu]-H_2NNO. The refined reaction energies and barriers of this reaction are given below in section 5.1. After observing [Z_2Cu]-HNNO, we started further simulations from this structure (setting # 7, see Table S1). By addition of NO_2, we observed the formation of N_2O, which is illustrated in Fig. S6.

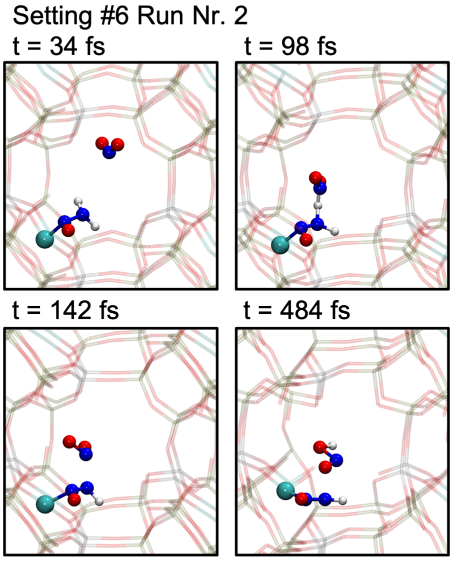

Figure S5. Hydrogen atom abstraction from [Z_2Cu]-H_2NNO by an NO_2 radical, forming [Z_2Cu]-HNNO. Upper left: start of the NMD simulation from [Z_2Cu]-H_2NNO and NO_2. Upper right: NO_2 abstracts the hydrogen atom from [Z_2Cu]-H_2NNO. Lower left: one HONO molecule is formed. Lower right: [Z_2Cu]-HNNO still persists.

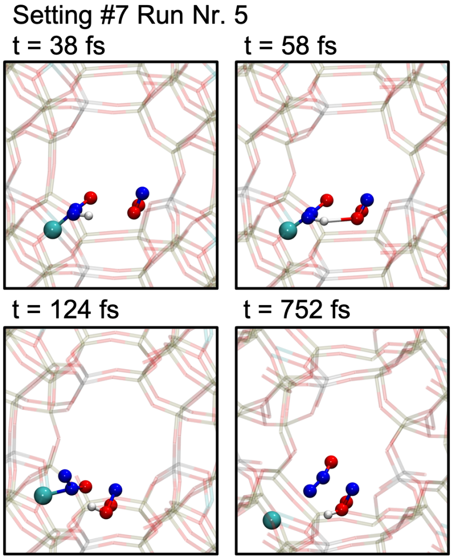

Figure S6. N_2O formation discovered using NMD simulations. Upper left: start of the trajectory from [Z_2Cu]-HNNO and NO_2. Upper right: hydrogen atom abstraction from [Z_2Cu]-HNNO by one of the NO_2 radicals. Lower left: [Z_2Cu]-NNO and one HONO molecule are formed. Lower right: After further 600 fs NMD simulation time, N_2O dissociates, releasing Cu(I).

2.4. Snapshots of the H_2O-shuttled mechanism of nitrosamine

NMD simulations starting from from [Z_2Cu]-H_2NNO with H_2O lead to the discovery of a water-shuttled tautomerization of nitrosamine, see Fig. S7.

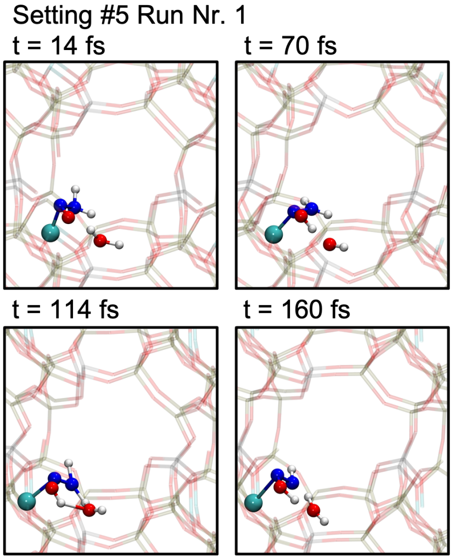

Figure S7. Snapshots of the water-shuttled formation of [Z_2Cu]-HNNOH as discovered by NMD simulations. Upper left: start of the trajectory from [Z_2Cu]-H_2NNO and H_2O. Upper right: deprotonation of water by Z_2Cu]-H_2NNO. Lower left: proton re-arrangement ongoing. Lower right: the completed reaction results in the formation of [Z_2Cu]-HNNOH.

2.5. 1,3-H-shift mechanism snapshots

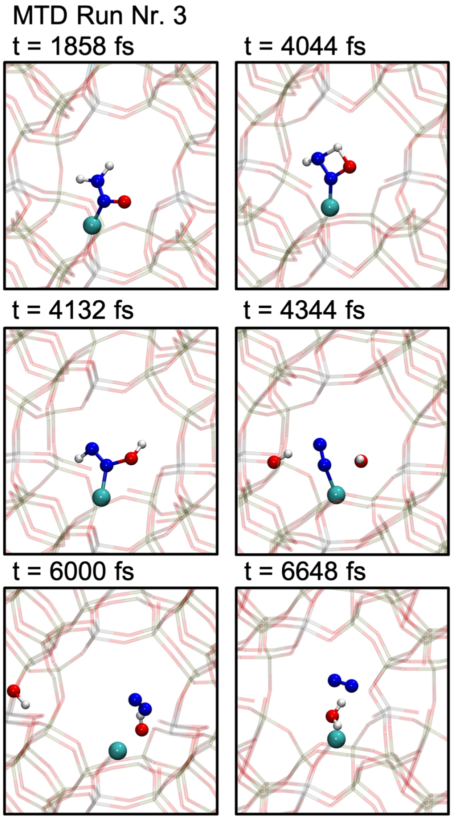

Figure S8. Snapshots of the 1,3 H shift from [Z_2Cu]-H_2NNO forming N_2 and H_2O discovered by RMSD-based metadynamics simulations. Upper left: [Z_2Cu]-H_2NNO before the 1,3 H shift. Upper right: Tautomerization product [Z_2Cu]-HNNOH as important intermediate of N_2 formation. Lower left: deprotonation by the zeolite framework and cleavage of hydroxy ion resulting in [Z_2Cu]-NN formation. Lower right: formation of H_2O and dissociation of the N_2 from the copper(I) atom.

2.6. Snapshot of water insertion into the zeolite framework

Initial disintegration reactions such as the insertion of a water molecule into the zeolite framework were observed. This reaction mechanism is in line with previous studies which show that water may lead to an irreversibe dealumination at high temperatures and large water concentrations. [17] In our simulations, a higher water concentration would be required to find the subsequent reaction steps of this degradation process.

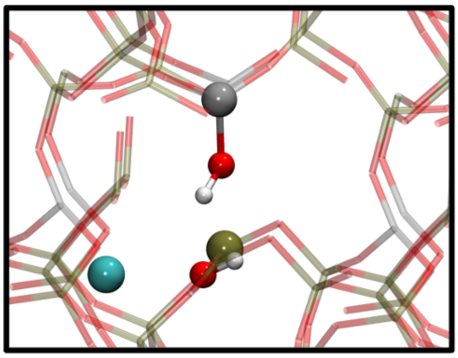

Figure S9. Snapshot of Setting #5, Run4 showing hydration of the zeolite framework as initial elementary reaction of dealumination under high temperatures.

3. Bond Detection

To enable automated reaction detection, we employed Wiberg bond orders, [18] monitoring bond dynamics throughout the nanoreactor MD simulations. To robustly distinguish true chemical transformations from transient interactions or thermal fluctuations, a two-threshold hysteresis scheme was applied. Specifically, a bond was considered formed when its bond order exceeded upper threshold BO_{upper}, and broken only when it dropped below the BO_{lower}. This hysteresis prevents the misidentification of high-amplitude vibrational motion as reaction events, ensuring that fleeting interactions are not mistaken for bond formation or cleavage. The thresholds were carefully selected to lie outside the range typically traversed by bond order fluctuations due to vibrations, as illustrated in Figure S10. This method enables consistent tracking of bond changes over time and forms the basis for constructing the reaction network from the nanoreactor trajectories.

Initially, a general set of bond order thresholds ($BO_{\mathrm{upper}} = 0.7$ for bond formation and $BO_{\mathrm{lower}} = 0.1$ for bond breaking) was applied uniformly across all atom pairs. However, the use of these values proved inadequate for describing coordination involving transition metals. In particular, Cu–O interactions exhibit inherently low bond orders, which led to underestimation of coordination events and misidentification of copper release from the zeolite framework. To address this, we introduced a custom threshold specifically for Cu–O atom pairs, enabling more accurate detection of Cu coordination and migration processes (see Table S2. for all adjusted thresholds). Notably, this adjustment was not required for Cu–N interactions, such as those involving Cu–NH_3 complexes, where bond orders remain sufficiently high for detection using the general thresholds. Furthermore, thresholds for Si–O and Al–O atom pairs were also modified to suppress spurious reactions that would otherwise result in the artificial detection of degradation reactions of the zeolite framework.

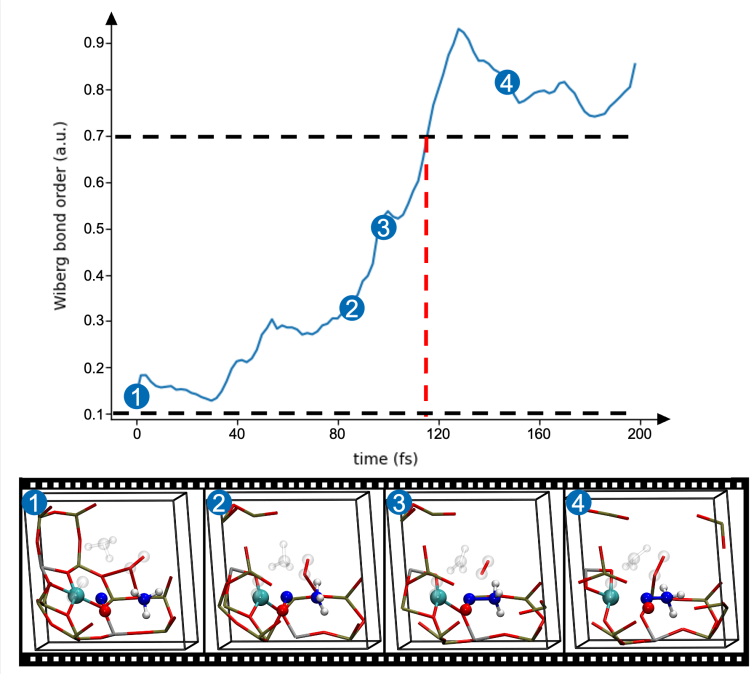

Figure S10. Bond order detection scheme for the example of the N-N bond formation of NO and NH_3 to H_3NNO. Above: time-evolution of the bond order and time steps. As soon as the upper threshold of 0.7 is crossed, a bond is considered formed. Below: Associated frameworks of the bond formation process: 1. Slightly interacting, but chemically unbonded NO and NH_3 2. and 3. Upon bond formation, the bond order shows some fluctuation in a short time span. 4. H_3NNO is stably formed, detected by crossing the 0.7 bond order value.

Table S2. Values for the thresholds BO_{upper} and BO_{lower} used to detect bond breaking and formation in general and for the Cu-O, Si-O, and Al-O atom pairs.

Atom pair	BO_{lower}	BO_{upper}
General	0.1	0.7
Cu-O	0.1	0.3
Si-O	0.01	0.17
Al-O	0.1	0.3

4. Reaction Network

In this work, 3050 unique species and 2810 unique reactions were discovered, are shown as blue nodes and black edges in Figure S11.

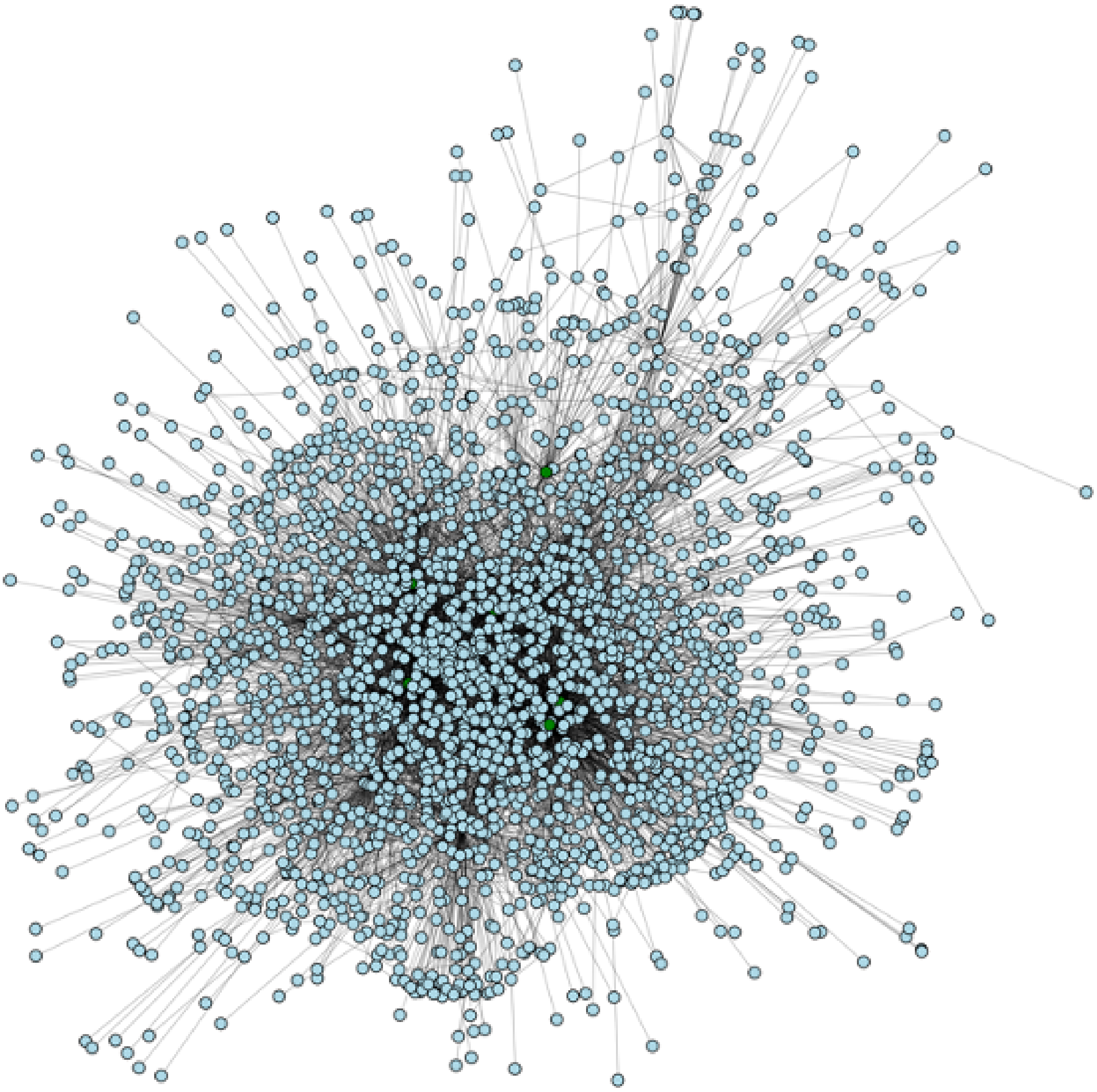

Figure S11. Reaction network including 3050 unique species (blue nodes) and 2810 unique reactions (black edges). Initial species: Z_2Cu, NO, NH_3, H_2O, and O_2 are shown as green nodes within the reaction network.

5. Refinement of Reaction Paths

Geometry optimization and transition state search were carried out using Chemshell [19] interfaced to Turbomole [6] and linked to the optimization library DL-Find [20]. The riper module with the PBE functional, a def2-SVP [21] basis set with a m5 grid and D3 dispersion correction [12] with Becke-Johnson damping [22] were used. For geometry optimization initial SCF-damping of 1.0, with a damping step of 0.05, and a minimum damping value of 0.1 and SCF convergence of 10^{-9} a.u. was used. For some structures, higher initial SCF-damping values of up to 25 are required to ensure SCF convergence. Settings regarding the periodic boundary conditions such as cell parameters, which were taken from the literature, [7] and Γ point were kept fixed for all optimizations. Reactions involving the adsorption of gas-phase molecules were calculated by considering the zeolite framework and gas-phase molecules separately. Ammonium formed during the process was positioned at its formation site within the zeolite framework to ensure charge neutrality and enable comparison between different adsorption structures. For gas phase molecules, free energies were obtained by using the rigid-rotor-harmonic-oscillator approximation as implemented in DL-find. For periodic systems, free energies were obtained by using the frozen-phonon approach (using harmonic approximation) implemented in Phonopy [23,24]. Two-point finite differences with a displacements of 0.02 Bohr were used for all atoms of the system (zeolite atoms and absorbed species) obtain numerical Hessians. All transition modes and translational movements of the lattice will be excluded from the thermodynamic analysis [25]. A strong initial SCF-damping value of 25, a damping step size of 0.1, and a minimum damping value of 0.5 were used to stabilize SCF convergence of the displaced structures. To ensure accurate vibrational analysis, the SCF convergence was set to 10^{-11} a.u. In order to obtain more accurate energies we computed single point calculations using the HSE06 [26,27] functional while keeping the computational settings of the optimization were performed.

5.1. N_2O formation mechanism

In Table S3 the potential and free energies of the refined species (A-D) for the N_2O formation mechanism provided in the main text are shown. In Figure S12 the potential energy refers to the sum of the potential energies of $[Z_2Cu]$-H_2NNO (A) and 2 NO_2 (separated). Including one NO_2 molecule in $[Z_2Cu]$-H_2NNO reduces the energy, forming a *pre-reactive complex* (A_{PRC}). The potential energy barrier of the transition state structure TS_{AB} is 7.7 kcal/mol concerning the energy of A_{PRC} and -0.6 kcal/mol with respect to the energy of A + 2 NO_2 (reference energy). The *post-reactive complex* (B_{PoRC}) could only be obtained by an IRC calculation and was not able to be optimized further. The separated structures of the formed $[Z_2Cu]$-HNNO (B, B_{cis}) could be optimized further. From structure B to B_{cis} a rearrangement of the HNNO radical is needed in order to be attacked once more. The *pre-reactive complex* B^*_{PRC} is lower by 11.7 kcal/mol in potential energy and is followed by the TS_{BC}. The potential energy barrier is 11.3 kcal/mol from the B^*_{PRC} and is -0.4 kcal/mol from separated molecules (B + NO_2). The reaction towards B^*_{PoRC} is strongly exothermic by -4.1 kcal/mol. If the HONO leaves the system the N_2O stays coordinated to the copper atom (C) after the release of the end product D is obtained.

Table S3. Potential energy (V), potential energy including zero point energy (V+ZPE), free energy (G) evaluated at 300 K and 670 K for the species involved in N_2O formation with PBE+D3(BJ)/def2-SVP in Hartree. V(HSE06) stands for the potential energy and G(HSE06, 670 K) for the free energy.Structures marked with * were taken from endpoints from the IRC. As they could not be further optimized, no corresponding ZPE or G values could be obtained.

Species	V	V+ZPE	G (300 K)	G (670 K)	V(HSE06)	G(HSE06, 670 K)
**Zeolite structures**						
A	-7064.630966	-7064.418228	-7064.454732	-7064.600557	-7064.920334	-7064.889925
A_{PRC}	-7269.392099	-7269.169658	-7269.212021	-7269.373937	-7269.639448	-7269.621286
TS_{AB}	-7269.385123	-7269.166546	-7269.207748	-7269.366954	-7269.627156	-7269.608987
B^*_{PoRC}	-7269.385852				-7269.633352	
B	-7064.000095	-7063.798435	-7063.833235	-7063.975824	-7064.278859	-7064.254588
B_{cis}	-7063.991004	-7063.790036	-7063.825180	-7063.968435	-7064.270809	-7064.248241
B^*_{PRC}	-7268.760389				-7268.995370	
TS_{BC}	-7268.756369	-7268.548987	-7268.589231	-7268.746350	-7268.977378	-7268.967359
C_{PoRC}	-7268.782166				-7268.988856	
C	-7063.421427	-7063.231909	-7063.268956	-7063.414923	-7063.697764	-7063.691260
D	-6879.039474	-6878.861743	-6878.892638	-6879.022157	-6879.349547	-6879.332230
**Gas phase molecules**						
NO_2	-204.738282	-204.729438	-204.749802	-204.780926	-204.705890	
NO_2H	-205.351186	-205.331471	-205.352483	-205.385537	-205.324143	
N_2O	-184.353499	-184.343722	-184.361979	-184.389754	-184.324464	
**Zeolite structures + gas phase molecules**						
A + 2 NO_2	-7474.107531	-7473.877105	-7473.954336	-7474.162409	-7474.332114	-7474.386992
A_{PRC} + NO_2	-7474.130381	-7473.899097	-7473.961823	-7474.154863	-7474.345338	-7474.369820
TS_{AB} + NO_2	-7474.123405	-7473.895984	-7473.957550	-7474.147880	-7474.333046	-7474.357520
B^*_{PoRC} + NO_2	-7474.124134				-7474.339242	
B + NO_2 + HNO_2	-7474.089564	-7473.859345	-7473.935520	-7474.142287	-7474.308892	-7474.361615
B_{cis} + NO_2 + HNO_2	-7474.080472	-7473.850945	-7473.927465	-7474.134898	-7474.300842	-7474.355268
B^*_{PRC} + HNO_2	-7474.111575				-7474.319513	
TS_{BC} + HNO_2	-7474.107555	-7473.880458	-7473.941714	-7474.131887	-7474.301521	-7474.325853
C_{PoRC} + HNO_2	-7474.133352				-7474.312999	
C + 2 HNO_2	-7474.123799	-7473.894852	-7473.973922	-7474.185997	-7474.346050	-7474.408248
D + 2 HNO_2 + N_2O	-7474.095345	-7473.868407	-7473.959583	-7474.182985	-7474.322297	-7474.409937

WILEY-VCH

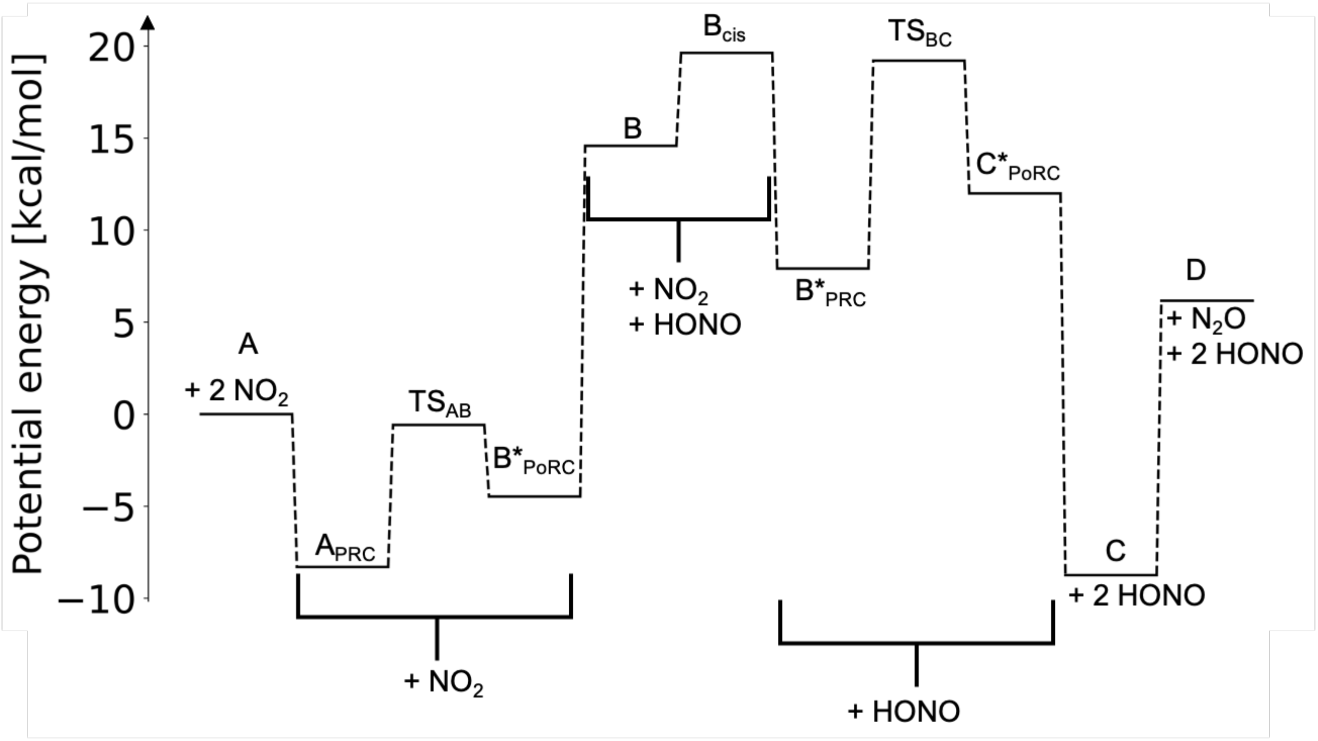

Figure S12. Potential energy profile with V(HSE06) of the N_2O formation in which [Z_2Cu]-H_2NNO + 2 NO_2 serves as a reference point and the gas phase molecules were added to maintain consistent potential energy.

5.2. H_2O-shuttled reaction mechanism

Figure S13 shows the potential energy profile of the water-shuttled HNNOH formation including the formation of a *pre reactive complex* (E). The energy of A and H_2O (separated) is used as reference. As a first step, the addition of one H_2O molecule to the system, i.e. close to [Z_2Cu]-H_2NNO, leads to a decrease in potential energy of 21.4 kcal/mol. The potential energy barrier of TS_{EF} is 9.8 kcal/mol with respect to the PRC (E) and -11.6 kcal/mol relative to the separated structures (A + H_2O). The 1,3 hydrogen shift TS_{AG} has a potential energy barrier of 38.6 kcal/mol. In comparison, the water-shuttled and 1,3 hydrogen shift mechanism results in a potential energy difference of 50.2 kcal/mol which makes the water-shuttled mechanism energetically more favorable.

Table S4. Potential energy (V), potential energy + zero point energy (V+ZPE), free energy (G) evaluated at 300 K and 670 K for the species involved in H_2O shuttled HNNOH formation in Hartree. V(HSE06) stands for the potential energy and G(HSE06, 670 K) for the free energy. Structures marked with * were taken from endpoint of the IRC. As they could not be further optimized, no corresponding *ZPE* or G values could be obtained.

Species	V	V+ZPE	G (300 K)	G (670 K)	V(HSE06)	G(HSE06, 670 K)
A	-7064.630966	-7064.418228	-7064.454732	-7064.600557	-7064.920334	-7064.889925
E	-7140.941698	-7140.704522	-7140.742782	-7140.896484	-7141.230037	-7141.184822
TS_{EF}	-7140.929239	-7140.721666	-7140.731226	-7140.880644	-7141.214504	-7141.165909
F*	-7140.931517				-7141.220273	
TS_{AG}	-7064.577529	-7064.369957	-7064.405978	-7064.550704	-7064.858842	-7064.832017
G	-7064.606705	-7064.394156	-7064.431084	-7064.577730	-7064.894367	-7064.865392
H_2O	-76.273273	-76.252569	-76.267895	-76.292232	-76.275655	-76.294614
A+H_2O	-7140.904239	-7140.670797	-7140.722627	-7140.892789	-7141.195989	-7141.184539
TS_{AG}+H_2O	-7140.850802	-7140.622526	-7140.673873	-7140.842936	-7141.134497	-7141.126631
G+H_2O	-7140.879978	-7140.646725	-7140.698979	-7140.869962	-7141.170022	-7141.160007

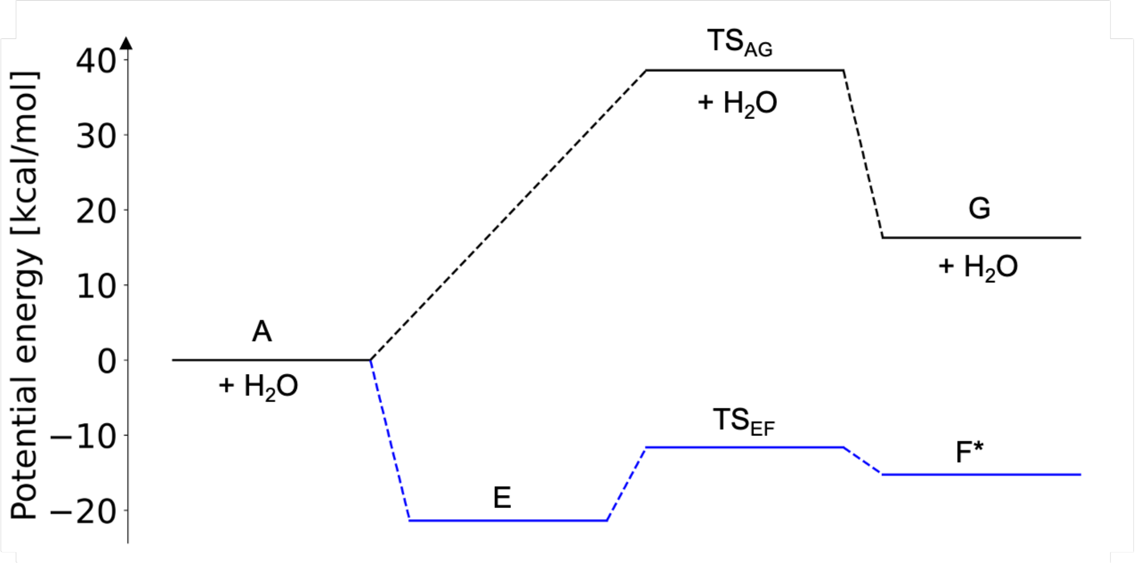

Figure S13. Potential energy profile of V(HSE06) for the water-shuttled HNNOH formation in which the [Z_2Cu]-H_2NNO (A) + H_2O serves as a reference point. The addition of the water molecule to the system decreases the energy (pre-reactive complex E) as the H_2O molecule is positioned between the oxygen and hydrogen atoms of [Z_2Cu]-H_2NNO and, therefore, two hydrogen bonds are formed.

References

[1] L.-P. Wang, A. Titov, R. McGibbon, F. Liu, V. S. Pande, T. J. Martínez, *Nat. Chem.* **2014**, *6*, 1044.
[2] A. M. Burow, M. Sierka, *J. Chem. Theory Comput.* **2011**, *7*, 3097.
[3] A. M. Burow, M. Sierka, F. Mohamed, *J. Chem. Phys.* **2009**, *131*.
[4] R. Łazarski, A. M. Burow, L. Grajciar, M. Sierka, *J. Comput. Chem.* **2016**, *37*, 2518.
[5] R. Łazarski, A. M. Burow, M. Sierka, *J. Chem. Theory Comput.* **2015**, *11*, 3029.
[6] Y. J. Franzke, C. Holzer, J. H. Andersen, T. Begušić, F. Bruder, S. Coriani, F. Della Sala, E. Fabiano, D. A. Fedotov, S. Fürst, S. Gillhuber, R. Grotjahn, M. Kaupp, M. Kehry, M. Krstić, F. Mack, S. Majumdar, B. D. Nguyen, S. M. Parker, F. Pauly, A. Pausch, E. Perlt, G. S. Phun, A. Rajabi, D. Rappoport, B. Samal, T. Schrader, M. Sharma, E. Tapavicza, R. S. Treß, V. Voora, A. Wodyński, J. M. Yu, B. Zerulla, F. Furche, C. Hättig, M. Sierka, D. P. Tew, F. Weigend, *J. Chem. Theory Comput.* **2023**, *19*, 6859.
[7] T. Anggara, C. Paolucci, W. F. Schneider, *J. Phys. Chem. C* **2016**, *120*, 27934.
[8] K. A. Feenstra, B. Hess, H. J. C. Berendsen, *J. Comput. Chem.* **1999**, *20*, 786.
[9] G. Bussi, M. Parrinello, *Phys. Rev. E* **2007**, *75*, 056707.
[10] S. Grimme, *J. Chem. Theory Comput.* **2019**, *15*, 2847.
[11] J. P. Perdew, K. Burke, M. Ernzerhof, *Phys. Rev. Lett.* **1996**, *77*, 3865.
[12] S. Grimme, J. Antony, S. Ehrlich, H. Krieg, *J. Chem. Phys.* **2010**, *132*.
[13] P. J. Hay, *J. Chem. Phys.* **1977**, *66*, 4377.
[14] A. Schäfer, H. Horn, R. Ahlrichs, *J. Chem. Phys.* **1992**, *97*, 2571.
[15] A. E. Reed, L. A. Curtiss, F. Weinhold, *Chem. Rev.* **1988**, *88*, 899.
[16] R. S. Mulliken, *J. Chem. Phys.* **1955**, *23*, 1833.
[17] M. Nielsen, A. Hafreager, R. Y. Brogaard, K. De Wispelaere, H. Falsig, P. Beato, V. Van Speybroeck, S. Svelle, *Catal. Sci. Technol.* **2019**, *9*, 3721.
[18] K. Wiberg, *Tetrahedron* **1968**, *24*, 1083.
[19] S. Metz, J. Kästner, A. A. Sokol, T. W. Keal, P. Sherwood, *WIREs Comput. Mol. Sci.* **2013**, *4*, 101.
[20] J. Kästner, J. M. Carr, T. W. Keal, W. Thiel, A. Wander, P. Sherwood, *J. Phys. Chem. A* **2009**, *113*, 11856.
[21] A. Schäfer, C. Huber, R. Ahlrichs, *J. Chem. Phys.* **1994**, *100*, 5829.
[22] S. Grimme, S. Ehrlich, L. Goerigk, *J. Comput. Chem.* **2011**, *32*, 1456.
[23] A. Togo, L. Chaput, T. Tadano, I. Tanaka, *J. Phys. Condens. Matter* **2023**, *35*, 353001.
[24] A. Togo, *J. Phys. Soc. Japan* **2023**, *92*.
[25] M. Rybicki, J. Sauer, *J. Chem. Theory Comput.* **2022**, *18*, 5618.
[26] J. Heyd, G. E. Scuseria, M. Ernzerhof, *J. Chem. Phys.* **2003**, *118*, 8207.
[27] A. V. Krukau, O. A. Vydrov, A. F. Izmaylov, G. E. Scuseria, *J. Chem. Phys.* **2006**, *125*.
